# IgG4-related mastitis characterized by skin thickening of the breast: a case report

**DOI:** 10.1186/s40792-023-01770-0

**Published:** 2023-11-01

**Authors:** Moe Itakura, Yoshiya Horimoto, Yumiko Ishizuka, Hiroko Onagi, Takuo Hayashi, Toshio Kawamoto, Junichiro Watanabe, Kotaro Iijima

**Affiliations:** 1 Department of Breast Oncology, Juntendo University Faculty of Medicine, 2-1-1 Hongo, Bunkyo-ku, Tokyo, 1113-0033 Japan; 2 Department of Human Pathology, Juntendo University Faculty of Medicine, 2-1-1 Hongo, Bunkyo-ku, Tokyo, 1113-0033 Japan; 3 Department of Internal Medicine and Rheumatology, Juntendo University Faculty of Medicine, 2-1-1 Hongo, Bunkyo-ku, Tokyo, 1113-0033 Japan

**Keywords:** IgG4-related disease, Mastitis, Skin thickening, Breast

## Abstract

**Background:**

IgG4-related diseases are characterized by marked infiltration and IgG4-positive plasma cells and fibrosis, and involve multiple organs. However, IgG4-related mastitis is rare. We report a case of mastitis associated with IgG4-related disease.

**Case presentation:**

A 78-year-old woman visited our hospital with a complaint of multiple subcutaneous nodules. A biopsy of a dorsal subcutaneous mass was performed but did not yield a definitive diagnosis. However, blood tests showed a high level of IgG4, thus she was referred to the department of collagen disease for further examination. Computed tomography (CT) showed a thickening of the skin of the right breast, and the patient was referred to our department. On physical examination, a large area of thickened skin was observed in the right breast without inflammatory breast cancer-like redness, and no mass was palpable. A needle biopsy was performed on an indistinct hypoechoic area in the breast, and she was diagnosed with mastitis associated with IgG4-related disease. Systemic steroid therapy was then administered and the symptoms of multiple skin nodules and mastitis improved.

**Conclusions:**

We reached the diagnosis based on a biopsy of the mammary gland enabling the patient to begin treatment for IgG4-related disease. This case was characterized by breast skin thickening, which is different from inflammatory breast cancer.

## Background

IgG4-related diseases are a relatively new disease concept proposed in the 2000s, and are characterized by marked infiltration of lymphocytes and IgG4-positive plasma cells and fibrosis [1, 2]. Single or multiple organ functions are irreversibly involved, and, typically accompanies autoimmune pancreatitis, sclerosing cholangitis, lacrimal and salivary gland inflammation, and IgG4-related kidney disease. On the other hand, IgG4-related mastitis is rare, and its clinical and imaging features are largely unknown [3, 4].

Herein, we report a case with mastitis as the main feature associated with IgG4-related disease which showed skin thickening.

## Case presentation

**Fig. 1:**
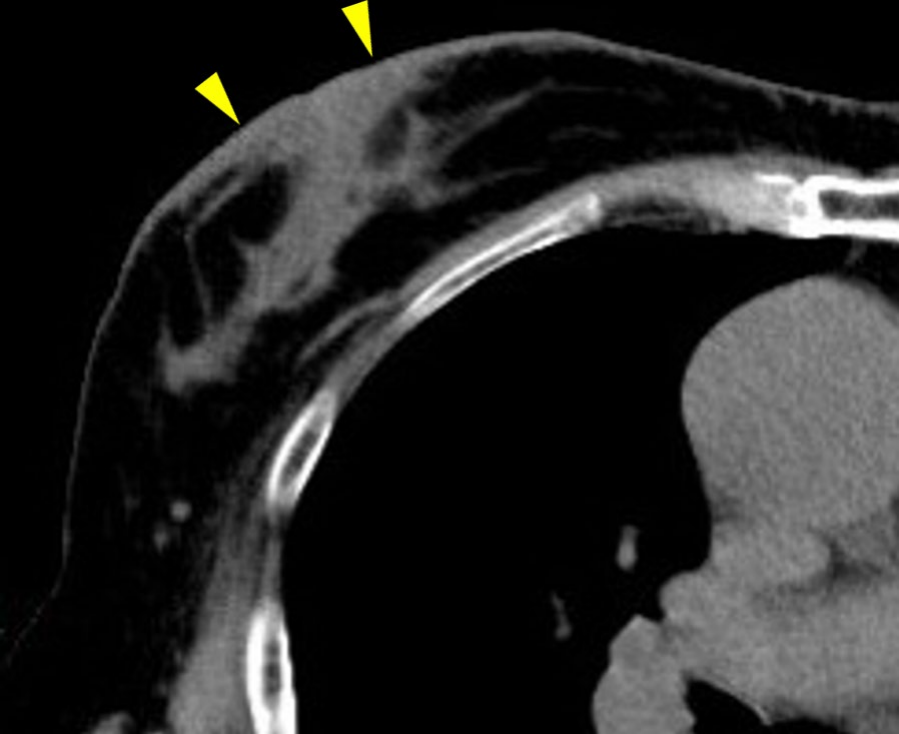
Computed tomography of the chest. Yellow arrowheads indicate skin thickening of the right breast

A 78-year-old woman visited the dermatology department of our hospital with a complaint of multiple subcutaneous nodules on her face, upper arms, and back. A needle biopsy was performed on a subcutaneous mass on her back. The histological assessment showed a lymphoid-like structure with slight infiltration of IgG4-positive plasma cells, but no definitive diagnosis was made. However, blood tests showed elevated serum IgG4 (947 mg/dL; normal range: < 121) and positive antinuclear antibody (320 units [normal < 19 units]), while anti-SSA/Ro antibody was negative, suggesting IgG4-related disease. Hence, she was referred to the department of collagen disease for further checkup. A whole-body computed tomography (CT) revealed a thickening of the skin of the right breast (Fig. 1), and she was referred to our department for further investigation. There were no obvious physical and imaging findings in other organs, such as the salivary glands or pancreas. Her medical history included cataracts, glaucoma, nasolacrimal duct obstruction, left heart enlargement, osteoarthritis of the knee, and reflux esophagitis. As for family history, an older sister had rheumatoid arthritis. There was no family history of breast or ovarian cancer.

**Fig. 2:**
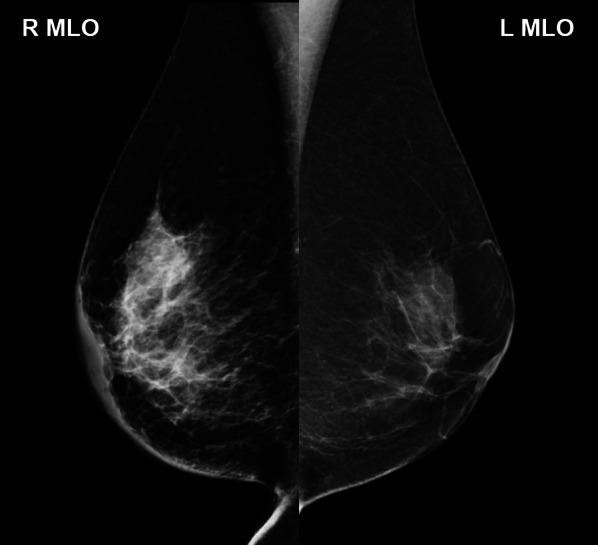
Findings from mammography. Thickening of the skin and Cooper’s ligaments of the right breast were observed with a high density of the mammary gland

**Fig. 3:**
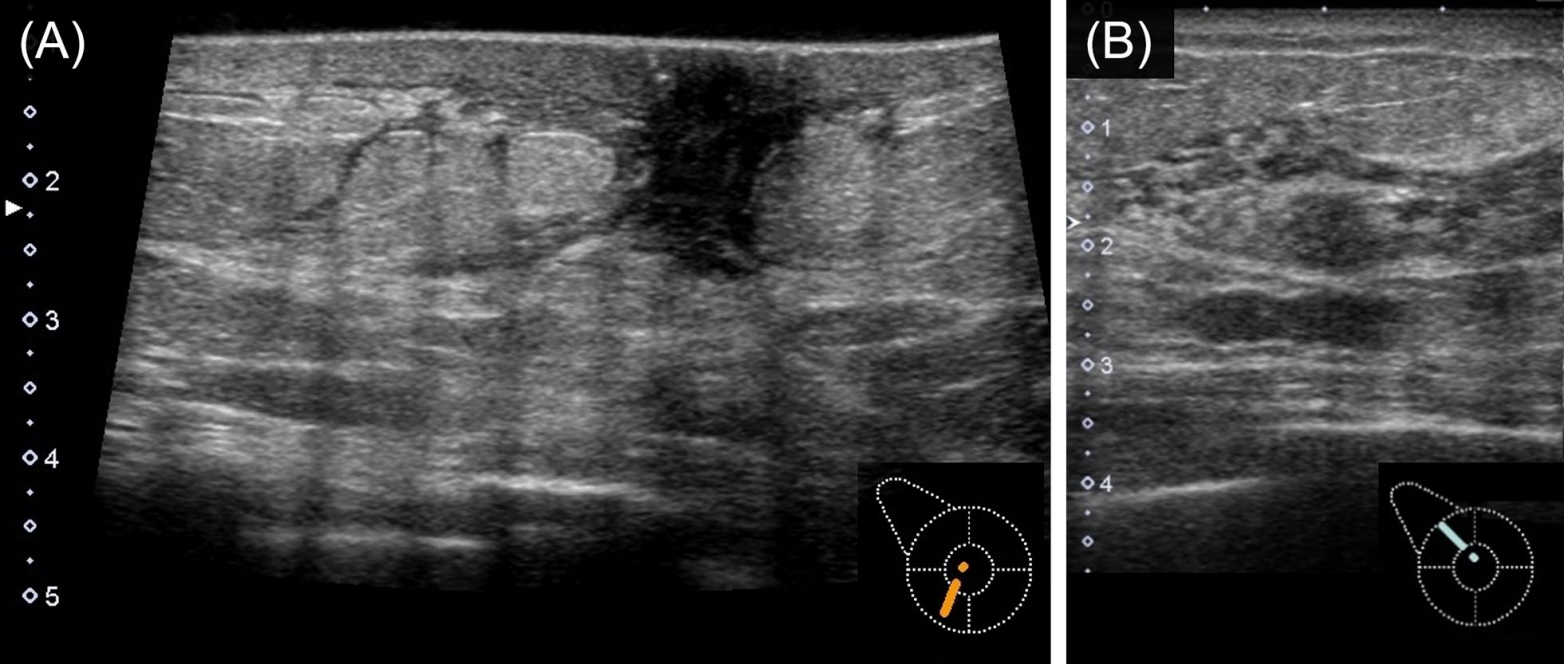
Ultrasonography of the right breast. **A** Thickening of the skin was observed at the lower lateral region. An increase in the echogenic level and thickening of the entire mammary gland was also seen compared to the contralateral breast. **B** A hypoechoic area with indistinct boundaries was seen within the mammary gland in the upper lateral area. It was accompanied by a slightly increased echogenicity of the surrounding fatty tissue

On physical examination, a large area of thickened skin, without redness, was observed on the lower part of the right breast. No breast mass or axillary lymph nodes were palpable. Mammography showed a thickening of the skin and areola in the right breast, and mammary gland density was higher than that of the left breast (Fig. 2). Ultrasonography revealed a thickening of the skin of the right breast (Fig. 3A), increased fat echogenicity, and development of intramammary vessels. Although a hypoechoic area was observed in the upper-outside of the mammary gland (Fig. 3B), there was no obvious mass formation. Thus, close follow-up imaging was deemed appropriate for the patient.

**Fig. 4:**
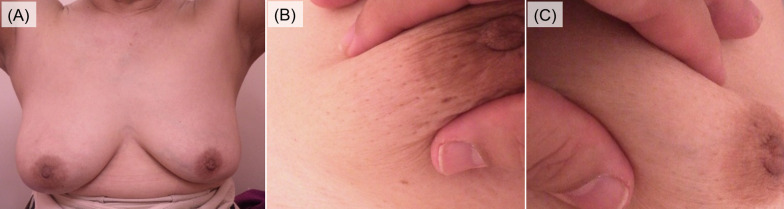
Findings of physical examination. **A** Slight swelling of the right breast. **B** Wrinkles were prominent when the right breast was pinched that were not seen in the left breast (**C**)

**Fig. 5:**
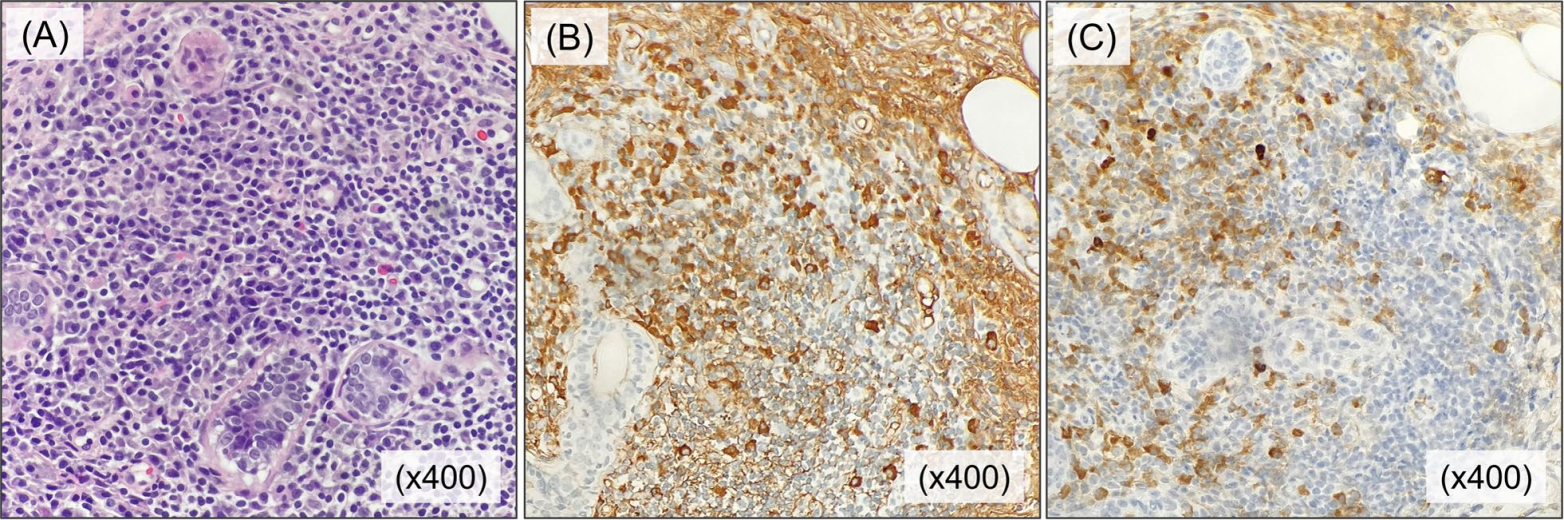
Histological findings of the biopsy specimen of the breast. Histological findings of the biopsy specimen are shown. **A** Hematoxylin and eosin staining revealed a marked lymphocyte infiltration surrounding the mammary ducts. **B** IgG4 and **C** IgG staining show marked infiltration of IgG4-positive plasma cells

Three months later, the skin findings were unchanged, with no erythema or obvious palpable mass, but the skin thickening remained in the right breast (Fig. 4A). Wrinkles were prominent when the right breast was pinched that were not seen in the contralateral breast (Fig. 4B, C). Ultrasonography also showed no obvious change in findings with no improvement; thus, a needle biopsy was performed on the hypoechoic area in the mammary gland. Histopathological findings are shown in Fig. 5. A high degree of IgG4-positive plasma cell infiltration was observed (200 cells/ high power field), with an IgG4/IgG rate of > 80%. Fibrosis was also prominent, although there was no specific florid fibrosis or phlebitis obliterans. No neoplastic lesions, including primary breast cancer, were observed. Considering other clinical symptoms and data, she was diagnosed with IgG4-associated mastitis.

After obtaining the diagnosis of IgG4-related disease, the patient was started on prednisolone (PSL; 30 mg/day). After initiation, the right brachial nodule reduced in size and other nodules gradually disappeared. She has been in remission for 6 months, with 15 mg/day of PSL to date.

## Discussion

According to previous reports, half of the cases of IgG4-associated mastitis were asymptomatic, i.e., mass without pain or redness, while the remainder had breast induration and mastalgia [3, 5, 6]. In the present case, the patient showed an asymptomatic breast mass with extensive skin thickening of the right breast. In general, inflammatory breast cancer should be ruled out when skin thickening is observed, however, in this case, there were no specific symptoms, e,g., diffuse erythema, edema, induration, or peau d'orange of the skin, related to inflammatory breast cancer. A core needle biopsy was performed on a hypoechoic lesion within the mammary gland, but it was not clear whether the same diagnosis would have been obtained if the thickened skin was biopsied.

The symptoms of IgG4-associated mastitis in this case were minor, and as far as mastitis alone is concerned, treatment may not have been necessary. IgG4-associated mastitis is not thought to increase the risk of breast cancer [7, 8]. However, IgG4-related disease basically involves multiple organs, and it is unlikely that the mammary gland is affected alone. If IgG4-related mastitis is initially diagnosed, other organs such as the hepatobiliary pancreas and otolaryngeal organs should be screened.

## Conclusions

We reached the diagnosis of IgG4-related disease based on a biopsy of the mammary gland allowing the patient to start treatment. This case was characterized by breast skin thickening, which is obviously different from inflammatory breast cancer. Mastitis with atypical symptoms should be carefully considered for biopsy given the possibility of this disease. We believe that the skin findings observed in this case should be noted as a finding of IgG4-related mastitis.

## Data Availability

Not applicable.

## Abbreviation

CT: Computed tomography
PSL: Prednisolone
